# GO based PVA nanocomposites: tailoring of optical and structural properties of PVA with low percentage of GO nanofillers

**DOI:** 10.1016/j.heliyon.2021.e06983

**Published:** 2021-05-07

**Authors:** K.M. Abu Hurayra–Lizu, Md. Wahidujjaman Bari, Fahmida Gulshan, Muhammad Rakibul Islam

**Affiliations:** a Department of Materials and Metallurgical Engineering, Bangladesh University of Engineering and Technology (BUET), Dhaka, Bangladesh; b Department of Physics, Bangladesh University of Engineering and Technology (BUET), Dhaka, Bangladesh

**Keywords:** Graphene oxide, PVA, Nanocomposite, Optical properties, Urbach energy

## Abstract

Graphene-based polymer composites are gaining interest as a modish class of substance that holds promising angles on diverse applications. In this work, Graphene Oxide (GO) based Polyvinyl Alcohol (PVA) nanocomposites (PVA-GO) have been prepared by employing a facile solution casting method. Low concentrations of GO nanofiller (0.25%, 0.50%, 0.75%, and 1.0%) were used and the result of the use of them over the distinct substantial characteristics of the nanocomposites was evaluated. The different features of the as-synthesized nanocomposites such as optical, structural, chemical, and thermal properties were identified by UV-Vis spectroscopy, X-ray diffraction (XRD), Fourier transform infrared spectra (FTIR), and Thermo-gravimetric analysis (TGA), respectively. From the structural analysis of the crystallinity of the nanocomposite it is evident that a reduction in crystallinity caused by the amalgamation of the GO nanofiller. FTIR study shows improved interaction between the GO nanofiller and PVA matrix. The incorporation of GO was found to reduce the optical band gap of the nanocomposite both for the direct and indirect transition. The Urbach energy of the nanocomposite increases with the increase of the GO concentration suggests the formation of localized states causing a reduction in the optical band gap. PVA-GO nanocomposites with improved and tunable physical properties synthesized from a simple and economic route may pave a new horizon for polymer-based optoelectronic devices.

## 1 Introduction

Polymer composites as sustainable materials are utilized in wide ranges of applications in the prospect of their ample variety of chemical compositions, room-temperature characteristics, applicability, their probable reusability together with the facility of construction and economy [1, 2]. Among this class of materials, polymer nanocomposites have gained considerable focus due to the increasing demand for more slender weight and exorbitantly performing substances, especially in automobile, aerospace, and defense research [3]. Combining traditional polymer matrix with nanostructured materials as reinforcements exhibit a wide range of extra-ordinary features due to their tiny size, distinctive shape, and large surface area [4, 5].

Graphene, a two-dimensional hexagonal carbon structure consists of sp2 bonded one-atom-thick planar sheet of carbon atoms are densely arranged crystal lattice of honeycomb formation [14]. Graphene exhibits some unique and outstanding features such as high thermal conductivity, excellent electron transport characteristics, and exclusive mechanical attributes, etc. [6, 7, 8, 9, 10]. Graphene displays a range of exotic intrinsic properties including high surface area, large aspect ratio, tensile strength (TS), EMI shielding ability, flexibility, transparency, etc. [6, 7, 8, 9, 10, 11, 12]. Due to these inherent properties, Graphene is considered a popular and preferable choice over other conventional nanofillers [13, 14].

However, pristine graphene is not considered as a suitable filler for polymers matrix as the bulk production of pristine graphene is time-consuming and costly. In contrast, graphene oxide, a derivative of graphene that contains different functional groups (hydroxyl, epoxy, carbonyl, etc), can make strong interaction with polymer and should be a more suitable nanofiller for organic polymers composite because of their easy, simple, and cost-effective technique [15, 16, 17]. Furthermore, GO contains different functional groups (hydroxyl, epoxy, carbonyl etc), that allow the formation of strong interaction with the polymer matrix. These functional groups in GO sheets make them hydrophilic, allowing them to readily swell and dissolve in water [18, 19, 20]. Synthesis of polymer nanocomposites with GO as nanofillers may have been converged on hydrophilic polymers such as poly (vinyl alcohol) (PVA) owing to their hydroxyl groups. As a hydroxyl-rich, water-soluble, biocompatible, and non-toxic polymer, PVA is extensively used in various applications for its availability, di-electric strength and promising optical feature [21, 22, 23]. Uniform dispersion of GO in the PVA matrix at a molecule level exhibits improvements in the mechanical properties, thermal stability, and electrical conductivity of the nanocomposite [24, 25, 26, 27]. Besides, the optical band gap of GO is about 1.5 eV, which indicates that it has efficient absorption in the ultraviolet (UV) zone and low absorption in the visible range [28, 29]. Thus incorporation of GO into the PVA matrix may occur significant changes in the optical properties of the nanocomposites with a considerable reduction of the bandgap and high absorbance value in the UV region. These optical property-tuning together with the structural changes due to the incorporation of GO have a direct consequence on the device performance.

Several studies have been performed to analyze the effect of GO nanofiller on the PVA matrix and in most of the cases, larger concentration of GO nanofiller was used [30, 31, 32, 33]. However, the effect of minute concentration of GO nanofiller on the structural and optical characteristics of PVA matrix has rarely been explored. Therefore, in this investigation, the influence of low GO concentration on the physical properties of the PVA-based nanocomposite has been explored. Nanocomposite synthesis techniques execute a pivotal function in tailoring the properties and performance of the nanocomposites. Among the available choices, solution casting is considered as a popular choice for the synthesis of nanocomposites because of its ease of processing, simple operation, and economic setup together with high-quality as-prepared sample [34, 35]. That is why in this work we used the solution casting method for the synthesis of PVA-GO nanocomposite and studied the structural, thermal and optical properties of the nanocomposites for a lower concentrations of GO.

## 2 Experimental procedure

### 2.1 Chemicals

Graphite powder and Polyvinyl Alcohol (PVA) powder were purchased from Qualikems Fine Chem. Pvt. Ltd. Sulfuric Acid (H_2_SO_4_) and Hydrogen Peroxide (H_2_O_2_) were acquired from Merck (Darmstadt, Germany). Potassium permanganate (KMnO_4_) was obtained from Merck, India. Sodium Nitrate (NaNO_3_) was obtained from Guangdong Chemicals. The chemicals were used without further purification. Deionized water (DI water) was used as a solvent in this study.

### 2.2 Preparation of GO

Modified Hummer's method was used to produce GO powder [36]. Firstly, graphite powder and sodium nitrate (NaNO_3_) with a ratio of 2:1 were dissolved in sulphuric acid (H_2_SO_4_) followed by stirring for 30 min. Then the solution was placed in an ice bath and potassium permanganate (KMnO_4_) was deliberately mixed such that the temperature of the solution was below 20 °C. After that, the ice bath was taken away and the temperature was raised to 35 °C and kept in stirring for 30 min until the mixture became pasty with brown-grey colour. The solution then heated for 65 °C for 1 h. De-ionized water was then added to the mixture and an exothermic reaction resulted, solution temperature was maintained at 98 °C for 15 min until it became brown. The solution was than further diluted with DI water and 30% H_2_O_2_ was added for complete removal of KMnO_4_. The solution then stirred at 70 °C for 2 h and filtered to obtain a solid cake which was washed, centrifuged, and finally ultrasonicated to form homogeneous dispersion of solute. Then, for 24 h it was heated at 70 °C to get hard solid Graphene oxide (GO). Grinding was performed to obtain desired GO powder.

### 2.3 Preparation of PVA-GO nanocomposites

PVA film with 0.25 wt.%, 0.50 wt.%, 0.75 wt.%, and 1.0 wt.% of nanofiller content was produced by the solution casting method. First, 1 g of PVA powder was added into 100 ml of DI water and maintained the temperature of the solution at 80 °C for half an hour to completely incorporate the solute. The mixture was then settled out to room temperature and ultrasonicated for one hour. Required amount of GO was dissolved in DI water and ultrasonicated for another hour to form homogeneous dispersion. This prepared GO solution was then added to the PVA solution periodically and was kept in continuous stirring with constant heat to obtain a viscous solution. This newly prepared solution was further ultrasonicated for 1 h and then transferred into a Polytetrafluroethylene (PTFE) coated pan. This cast solution was then placed in an oven at 70 °C for 24 h to dry and to acquire a thin film of PVA-GO nanocomposites. To compare the properties, a pure PVA film was also prepared using the identical procedure.

### 2.4 Characterization

X-ray diffraction of PVA-GO nanocomposites was performed by a two-circle (2θ-θ) X-ray powder diffractometer (X'Pert XRD PRO PW 3040). Fourier-transform infrared spectroscopy (FTIR) of PVA-GO nanocomposites was performed by Cary-630 FTIR instrument by Agilent Technologies. The Q50 TGA instrument was used to evaluate the thermal stability of the prepared composite materials through the thermogravimetric analysis (TGA) process in an inert atmosphere. TGA was performed in nitrogen medium and a heating rate of 10ºC/min was used to raise the temperature from room temperature to 650 °C. UV-Vis spectroscopy was performed by HALO DB-20S UV-Vis double beam spectrometer to study the optical properties of the nanocomposite.

## 3 Results & discussion

### 3.1 Structural properties

**Figure 1:**
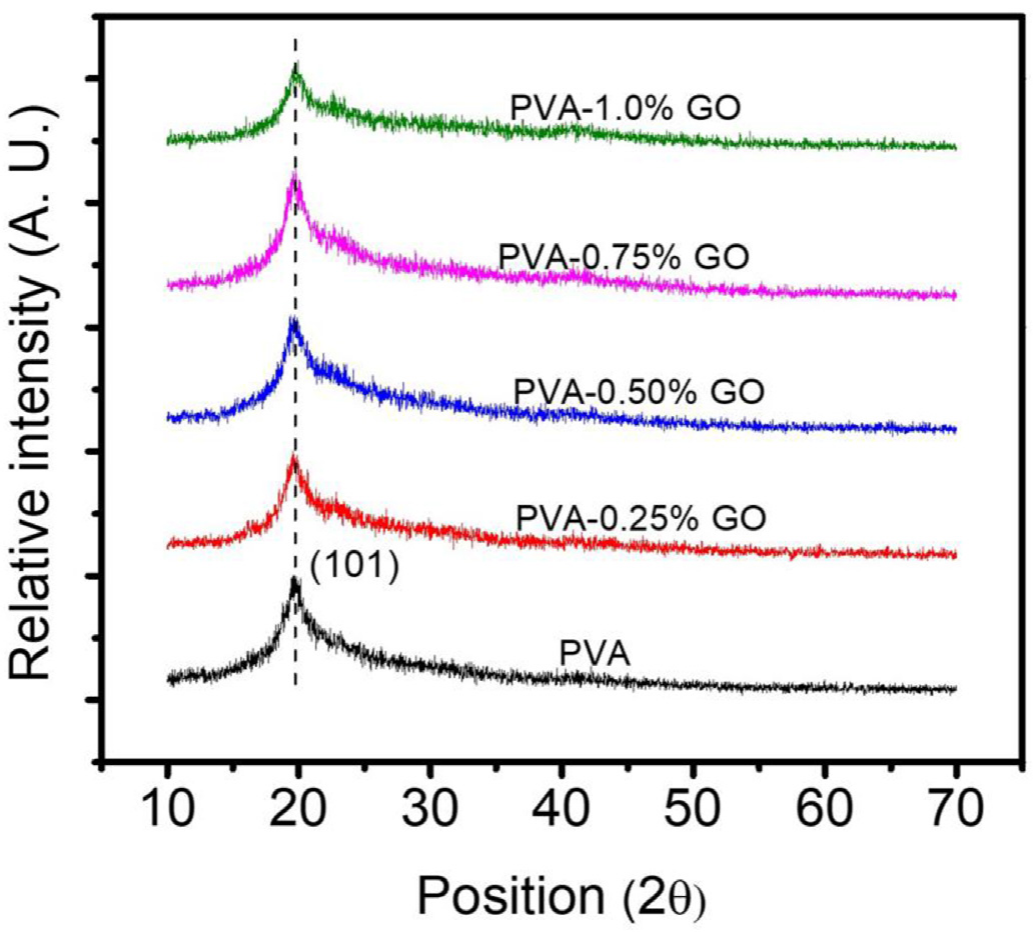
XRD analysis of pure PVA and PVA-GO nanocomposites.

The XRD spectrum of as-prepared PVA/GO nanocomposite for different concentrations of GO loading is presented in Figure 1. PVA shows a characteristic peak at 19.68^o^ corresponding to the (101) crystalline phase of the polymer [37]. When PVA is reinforced with GO, all the samples have similar curves and no additional peak for GO was present in the XRD data. This suggests that the GO nanofillers do not change the crystalline structure of the PVA matrix. This also indicates that at the molecular phase, the GO nanofillers are well dispersed within the PVA matrix. The intensity of the (101) peak was found to be reduced with the concentration of the GO nanofiller suggesting a reduction in the crystalline quality of the nanocomposite. The unison packing of the number of PVA chains is evident from the acuteness of the (101) diffraction. And a decrease in intensity of this peak due to the incorporation of GO may be attributed to the restacking tendency of GO nanofillers [38].

At low filler loading, the nucleation of GO dominates over the hydrogen bonding between GO and PVA. Whereas with the increase of the amount of filler content the hydrogen bonding between the polymer matrix and nanofiller becomes stronger compared to the nucleation of GO. For quantitative analysis, the effect of GO content on the relative crystallinity of the nanocomposite was estimated. The relative crystallinity was measured using the equation [39]:

(1) $$X_{c}=\left(A_{c}/A_{t}\right)\times 100\%$$

where *A*_*c*_ and *A*_*t*_ are areas under crystalline peaks and total area under the curve respectively. The degree of crystallinity of the nanocomposites was calculated by substituting the corresponding values of *A*_*c*_ and *A*_*t*_ obtained from the (101) diffraction peak of the respective XRD curve into the above Eq. (1). Table 1 represents the corresponding relative crystallinity of the nanocomposites for different filler concentrations. Declination in the crystallinity of the nanocomposite was observed with the increase of GO nanofiller concentration. Incorporation of GO may introduce disorder into the polymer matrix and interrupt the packing of the polymer chain resulting in a decrease of the crystallinity of the nanocomposite.

**Table 1:** Relation between Direct band gap, indirect band gap (E_g_), Urbach energy (E_u_) and Degree of Crystallinity (*X*_*c*_) values for pure PVA and PVA-GO nanocomposites.

Sample	Direct band gap (eV)	Indirect band gap (eV)	Urbach Energy (eV)	Crystallinity (%)
PVA	5.55	4.82	0.36	34
PVA-0.25% GO	5.45	4.69	0.44	30
PVA-0.50% GO	5.37	4.49	0.67	27
PVA-0.75% GO	5.32	4.37	0.52	25
PVA-1.0% GO	5.15	4.27	0.79	24

### 3.2 Chemical properties

**Figure 2:**
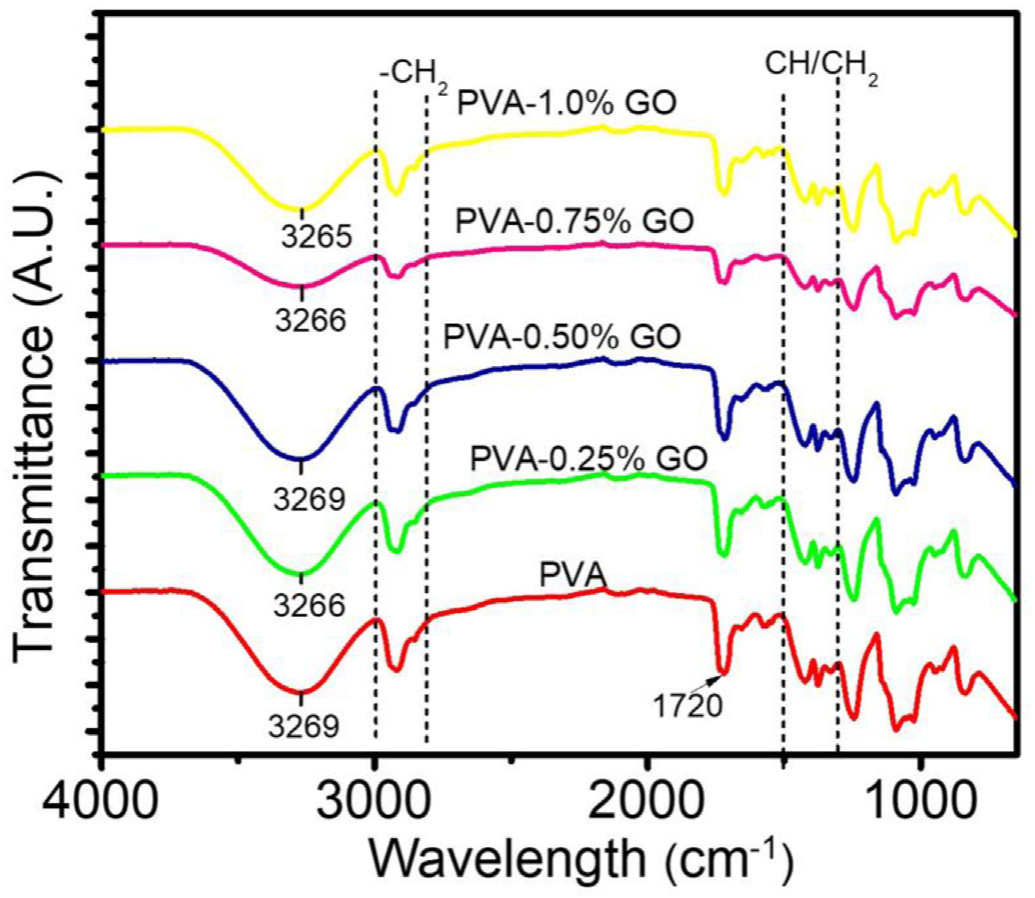
FTIR spectra for Pure PVA and PVA-GO nanocomposites.

To assess the interaction of GO nanofiller with PVA, the FTIR spectra of the nanocomposites were analyzed (Figure 2). In the FTIR spectrum of PVA, a broad absorption peak, extended between 3000 cm^−1^ to 3700 cm^−1^ is observed and can be attributed to –OH symmetrical stretching vibrations and this substantiate the presence of intense intermolecular hydrogen bonding between the nanofiller and matrix. The absorption band between 2800 cm^−1^ to 3000 cm^−1^ and 1300 cm^−1^ to 1500 cm^−1^ suggesting the presence of to –CH2 stretching and –CH/CH2 deformation vibrations, respectively. The peak at 1720 cm^−1^ PVA Corresponds to the stretching vibrations of C=O group [40, 41]. With the incorporation of GO content in the nanocomposite, the –OH stretching vibration peak was found to be moved towards the lower wavenumber. When GO has added the hydrogen bonding in the hydroxyl group of PVA got dissociated and hydrogen bond forms between PVA and GO. The augmentation of GO nanofillers might be the effect of the hydrogen bond degeneration in the OH groups of the PVA matrix. The diminution of hydrogen bonding in the –OH group of PVA generates the arrangements of hydrogen bond amidst PVA chain and GO and is the characteristic intensification key factor [41, 42].

### 3.3 Thermal properties

**Figure 3:**
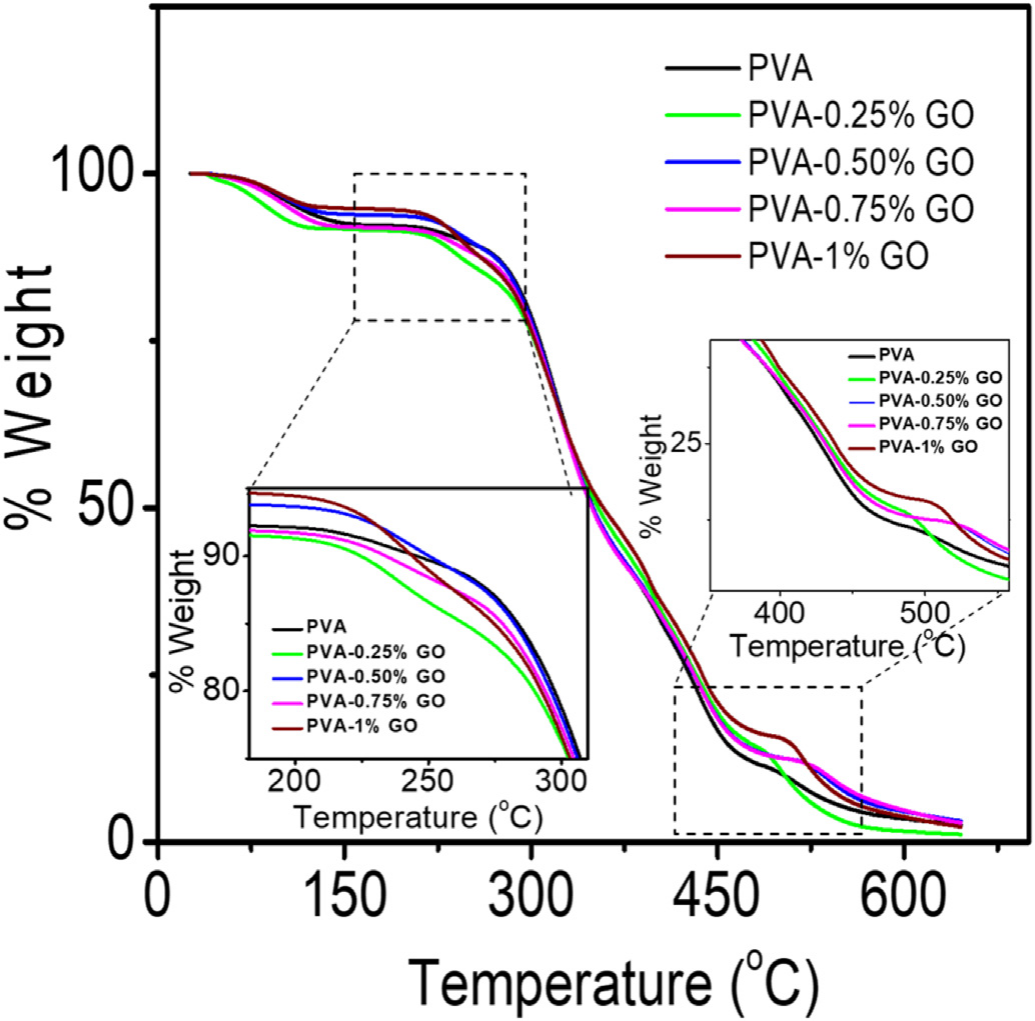
TGA curves for pure PVA and PVA-GO nanocomposites.

The thermal properties of PVA and PVA-GO nanocomposite's were evaluated by TGA and the corresponding data were presented in Figure 3. The neat PVA displays thermal decomposition via double stages which is evident from the TGA study. The side chains and the main chain of the PVA disintegrate at approximately between 200 °C to 350 °C, and 400 °C–550 °C which signifies the decomposition temperature, respectively [39]. Upon examining the TGA plot, not much significant change in the thermal behavior of PVA-GO nanocomposites was observed. The onset of decomposition temperatures, both in the first and second stage, was found to be varied due to the incorporation of GO content. This indicates that there is a strong interaction between PVA and GO nanofillers at the interface due to the formation of hydrogen bonds, and, because of this, close to the interfaces the polymer chain's mobility has reduced [38].

### 3.4 Optical properties

**Figure 4:**
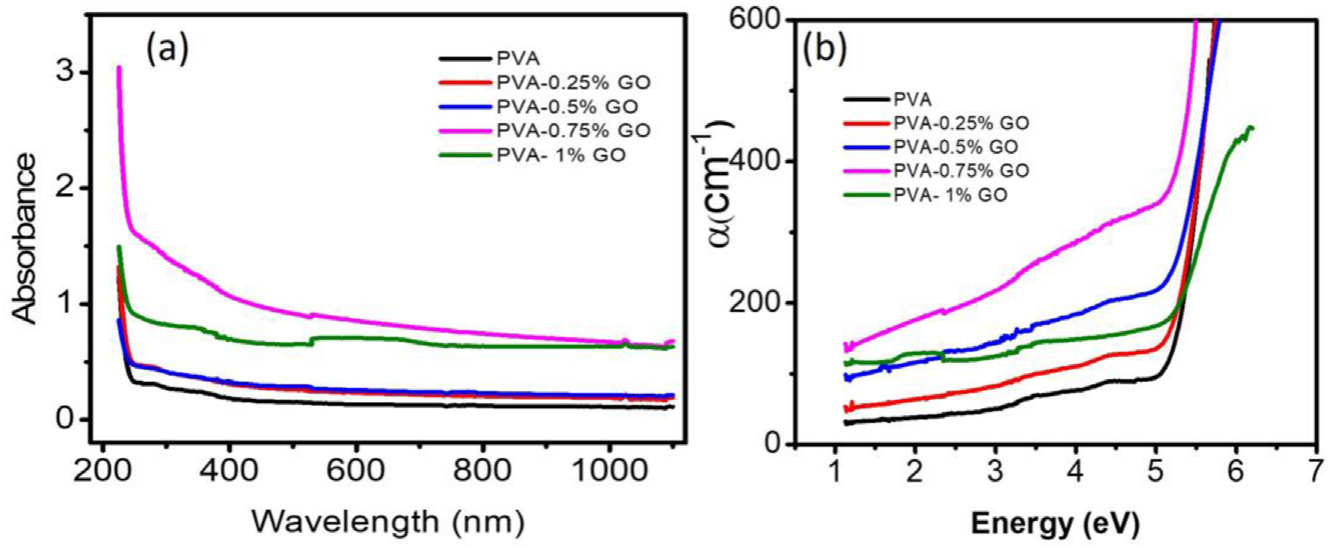
(a) Absorbance spectra and (b) calculated absorption co-efficient for pure PVA and PVA-GO nanocomposites.

Figure 4(a) displays the effect of GO content on the optical absorption of the PVA-GO nanocomposites observed from UV-vis spectroscopy. Optical absorbance was found be increases with increasing GO content in the nanocomposite. This data is useful to interpret information relating to the optical energy band gap and electronic band structure of the polymer nanocomposites. Absorption coefficient, α is evaluated using the Beer-Lambert's formula [43]:

(2) $$\alpha =\left(2.303I/I_{o}\right)/d=\left(2.303A\right)/d$$

where *d* is the sample thickness and *A* is the absorbance. Figure 4(b) shows the variation of absorbance obtained using Eq. (2) as a function of wavelength for the PVA-GO nanocomposite. The optical absorption of the nanocomposites was found to increases in the UV with the increase of GO concentration. The observed increase of the UV shielding performances of the PVA-GO films with the incorporation of GO may be attributed to two factors. Firstly, the uniform dispersion of GO nanoflake allows effective absorption of the UV light and thereby converts them into heat. Secondly, the UV light may be scattered from the interface between the PVA/GO interfaces created by the hydrogen bonding [39].

The optical energy gap of the materials can be determined from this absorbance data using Tauc's expression [43]:

(3) $$\alpha h\nu =\beta {\left(h\nu -E_{g}\right)}^{n}$$

where *h* = Planck's constant, *β* is a constant depending on the specimen structure, *E*_*g*_ is the optical band gap energy, and the exponent n is an empirical index. The value of n in Eq. (3) depends on the nature of the electronic transition of the absorption. They can take the values equal to ½, 3/2, 2, and 3 for direct allowed, direct forbidden, indirect allowed, and indirect forbidden, respectively.

**Figure 5:**
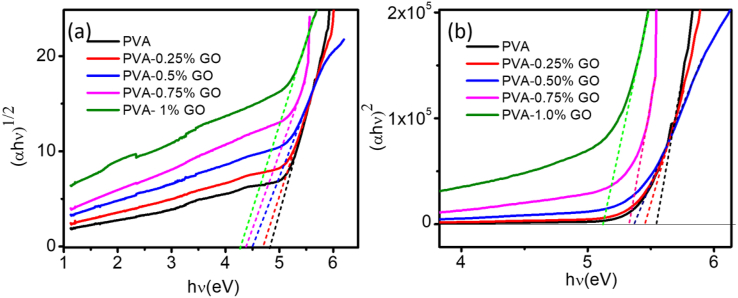
(a) Determination of E_g_ using Tauc's expression. (b) Relation between GO concentration in nanocomposites and E_g_.

To determine the band gap values *(αhν)*^*1/n*^ were plotted against *hν*. The best fit for the present optical data is observed for *n = 2*, which is represented in Figure 5(a). The extrapolation of linear portions of these plots to the energy axis, yields the indirect band gap of the samples. The direct band gap of the nanocomposites was estimated from the *(αhν)*^*2*^ vs *hν* plot and is presented in Figure 5 (b). The estimated values of the corresponding direct and indirect band gap of the PVA-GO nanocomposites for different concentrations of GO nanofillers are demonstrated in Table 1.

**Figure 6:**
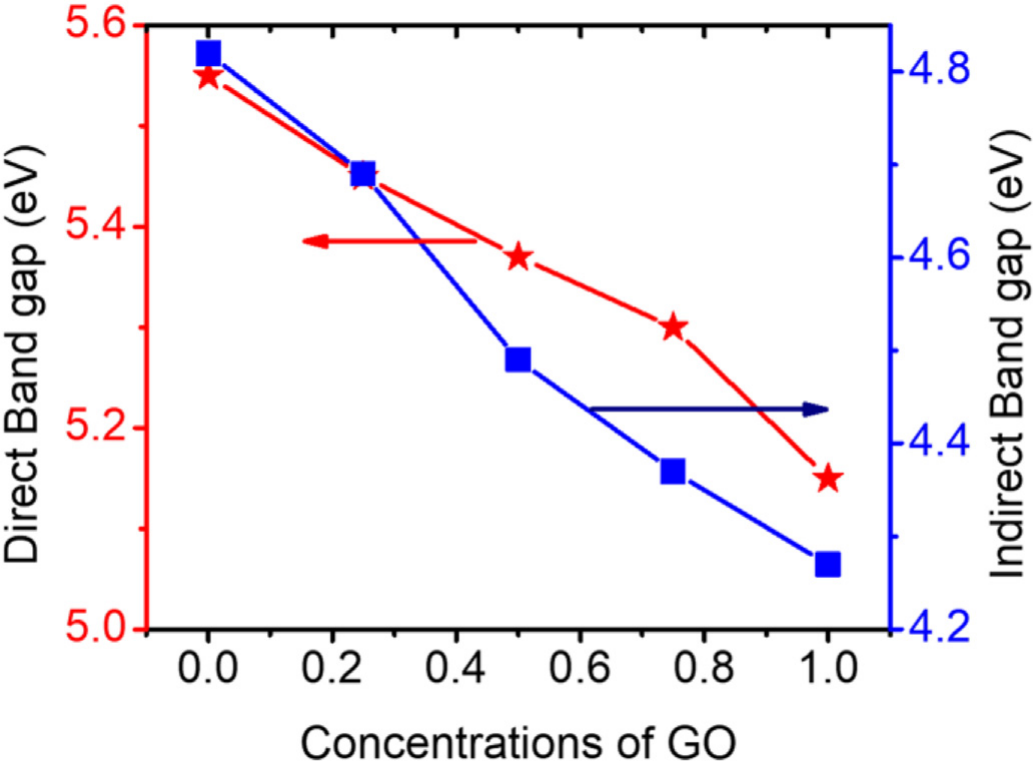
Variation of the direct and indirect band gap of the PVA-GO nanocomposite with the GO concentration.

Figure 6 depicts the variation of the direct and indirect band gap of the PVA-GO nanocomposite as a function of GO content. Both the direct and indirect band gap were composed to be varied linearly with the GO content. Due to the incorporation of GO new energy levels may form in between the conduction and valance band and thereby reduces the band gap of the nanocomposite [33, 44].

**Figure 7:**
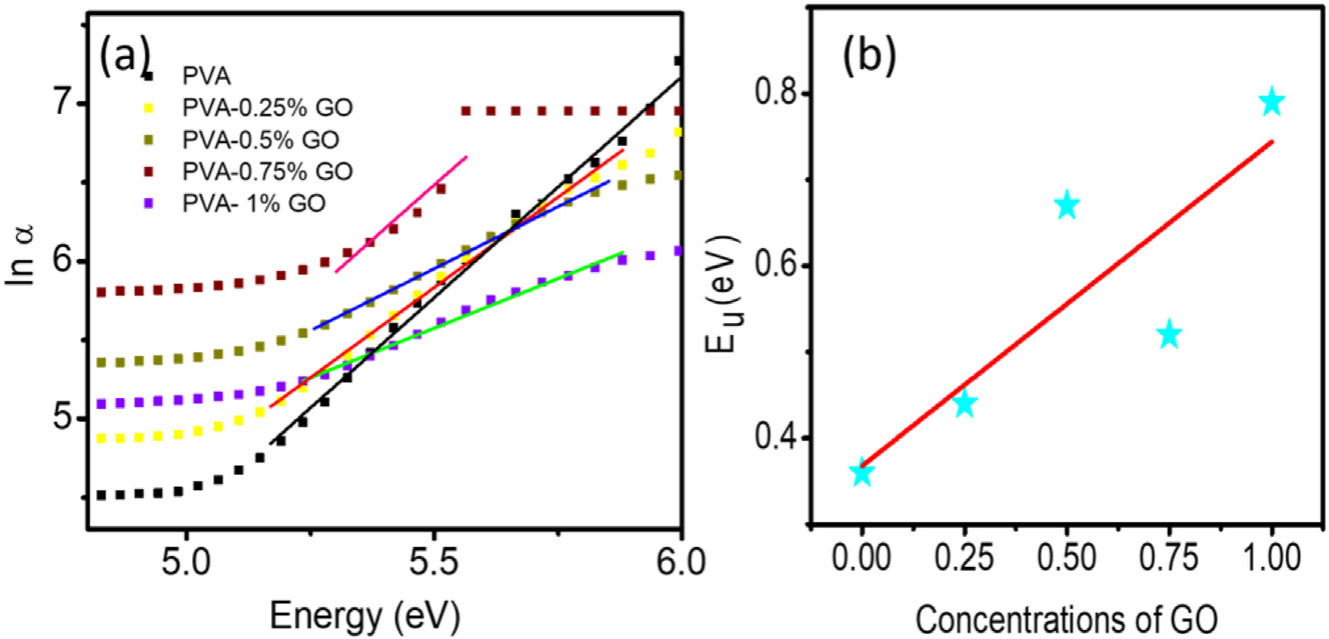
(a) Urbach Energy determination for pure PVA and PVA-GO nanocomposites. (b) Linear dependence between E_u_ and GO concentrations in nanocomposites.

In an optical transition, an electron from the valance band captivates photon and move to the valance band across the band gap. During this transition, the electron may experience disorders caused by defect centers or thermal vibration. This disorder creates density of states tailing into the forbidden energy gap. The breadth of this tail, called the Urbach tail, suggests the presence of defect levels in the forbidden gap between the valence and conduction bands. The energy associated with this tail is referred to as Urbach energy (E_u_) which can be determined by the relation [45].

(4) $$\alpha \left(\nu \right)=\alpha_{\text{o}}\left(\nu \right)\exp\left(h\nu /E_{u}\right)$$

where α_o_ (ν) is a constant. Figure 7(a) demonstrates a plot of the natural logarithm of the absorption coefficient *α(ν)* as a function of the photon energy (*hν*). As can be seen from Eq. (4), the *E*_*u*_ values are determined from the inverse of the slopes of the straight-line portion of the graphs and are summarized in Table 1. The *E*_*u*_ value found to be increased gradually from 0.356 eV for pure PVA to 0.793 eV for PVA-1% GO. This trend of the increasing of the defect into the PVA matrix matches with the observed XRD data where the intensity of the dominant diffraction peaks reduces with the increase of the GO concentration.

Urbach energy is a measure of the amorphousness due to inhomogeneous disorders in the materials [46]. An increase in Urbach energy suggests an increase of the amorphous portion in the materials [47]. The increase in Urbach energy (*E*_*u*_) due to the incorporation of GO can therefore be attributed to the creation of ionic complexes, disruption, and deformities in the host polymer due to increased interaction between various surface oxygenated groups on GO and PVA.

The increase of Urbach energy also suggests that the incorporation of GO introduces localized states within the forbidden energy bandgap [48]. The presence of such localized states may also act as trap center for charge carriers and can favor the photocatalytic degradation process [48, 49].

## 4 Conclusion

In conclusion, PVA-GO nanocomposite with low concentrations of GO nanofiller was synthesized by solution casting method. FTIR analysis represents the formation of hydrogen bonding between GO nanofiller and polymer matrix. GO was found to reduce both the direct and indirect optical band gap of the nanocomposite. An increase in Urbach energy of the nanocomposite was also observed suggesting formation of localized states due to the incorporation of GO causing the reduction of band gap. PVA-GO nanocomposites with tunable optical properties synthesized from a simple and economic route may pave a new horizon for polymer-based optoelectronic devices.

## Declarations

### Author contribution statement

K.M. Abu Hurayra–Lizu: Conceived and designed the experiments; Performed the experiments, Analyzed and interpreted the data; Wrote the paper.

Md. Wahidujjaman Bari: Analyzed and interpreted the data.

Fahmida Gulshan: Contributed reagents, materials, analysis tools or data.

Muhammad Rakibul Islam: Conceived and designed the experiments; Analyzed and interpreted the data; Contributed reagents, materials, analysis tools or data; Wrote the paper.

### Funding statement

This research did not receive any specific grant from funding agencies in the public, commercial, or not-for-profit sectors.

### Data availability statement

Data will be made available on request.

### Declaration of interests statement

The authors declare no conflict of interest.

### Additional information

No additional information is available for this paper.
